# Host-Guest Interaction of Cucurbit[8]uril with *N*-(3-Aminopropyl)cyclohexylamine: Cyclohexyl Encapsulation Triggered Ternary Complex

**DOI:** 10.3390/molecules23010175

**Published:** 2018-01-15

**Authors:** Yu Xia, Chuan-Zeng Wang, Mengkui Tian, Zhu Tao, Xin-Long Ni, Timothy J. Prior, Carl Redshaw

**Affiliations:** 1 Key Laboratory of Macrocyclic and Supramolecular Chemistry of Guizhou Province, Guizhou University, Guiyang 550025, China; tmxy94@163.com (Y.X.); 13639028944@163.com (C.-Z.W.); tianmk78@126.com (M.T.); gzutao@263.net (Z.T.); 2 Department of Chemistry, School of Mathematics and Physical Sciences, University of Hull, Hull HU6 7RX, UK; t.prior@hull.ac.uk

**Keywords:** cucurbit[8]uril, cycloalkane groups, host-guest interaction, ternary complex, X-ray structure

## Abstract

The host-guest interaction of a series of cyclohexyl-appended guests with cucurbit[8]uril (Q[8]) was studied by ^1^H NMR spectroscopy, isothermal titration calorimetry (ITC), and X-ray crystallography. The X-ray structure revealed that two cycloalkane moieties can be simultaneously encapsulated in the hydrophobic cavity of the Q[8] host to form a ternary complex for the first time.

## 1. Introduction

In supramolecular chemistry, the encapsulation of two or more guest molecules into a host cavity has attracted increasing attention because of their potential wide application in a variety of areas [1,2], such as in molecular reactive containers [3,4], molecular recognition [5], supramolecular polymers [6], and in fluorescent materials [7]. Among the various artificial macrocyclic host receptors, cucurbit[8]uril (Q[8] or CB[8]) [8,9], which is a large homologue of the Q[n] family, is particularly attractive because it is large enough to accommodate simultaneously two hetero- and homo-aromatic guests in its cavity to form a 1:2 ternary complex in aqueous solution. For example, a number of Q[8]-based supramolecular assemblies and architectures has been successfully constructed by Kim et al. [2], with the Q[8] host generally encapsulating one electron-donor molecule and one electron-acceptor molecule inside the cavity to form a stable 1:1:1 ternary complex. Moreover, Kaifer et al. demonstrated the recognition of neurotransmitters such as dopamine by using Q[8] host-stabilized charge transfer interactions [10]. Ramamurthy et al. discovered that the presence of a Q[8] cavity-triggered ternary complex can be beneficial for photodimerization reactions [11]. In other work, Urbach exploited the potential of Q[8]·MV^2+^ (MV is a methyl viologen) to recognize *N*-terminal tryptophan in aqueous solution [12]. More recently, Zhang [13], Scherman [14], and Li [15] have reported a number of smart supramolecular polymers based on Q[8] host cavity-stabilized charge transfers or π–π interactions. It should be noted here that at least one aromatic group of each guest molecule was situated in the cavity of the Q[8] in the above-mentioned works.

In 2008, Kim et al. reported that alkyl chains can adopt a U-shaped conformation when bound in the cavity of Q[8] [16]. Later, our group discovered that not only can two alkyl groups be encapsulated in the same Q[8] host instead of two aromatic groups [17], this can also further result in the ternary complex forming supramolecular polymers [18]. Herein, as part of our continued interest in Q[8] ternary complexes, we wondered whether two cyclohexyl groups could be encapsulated in the same Q[8] host cavity. From a structural viewpoint, the cyclohexyl moiety has a similar structure in terms of sterics that is more reminiscent of an aromatic molecule rather than an alkyl group.

## 2. Results and Discussion

**Scheme 1:**
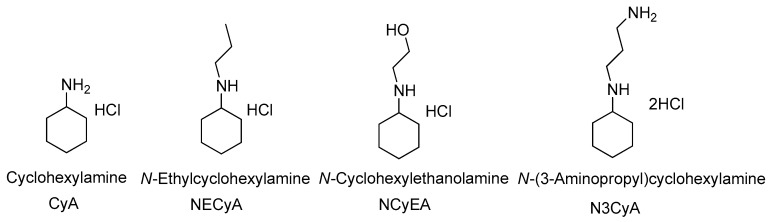
Chemical structures of cyclohexylamine derivatives (hydrochloride salts).

**Figure 1:**
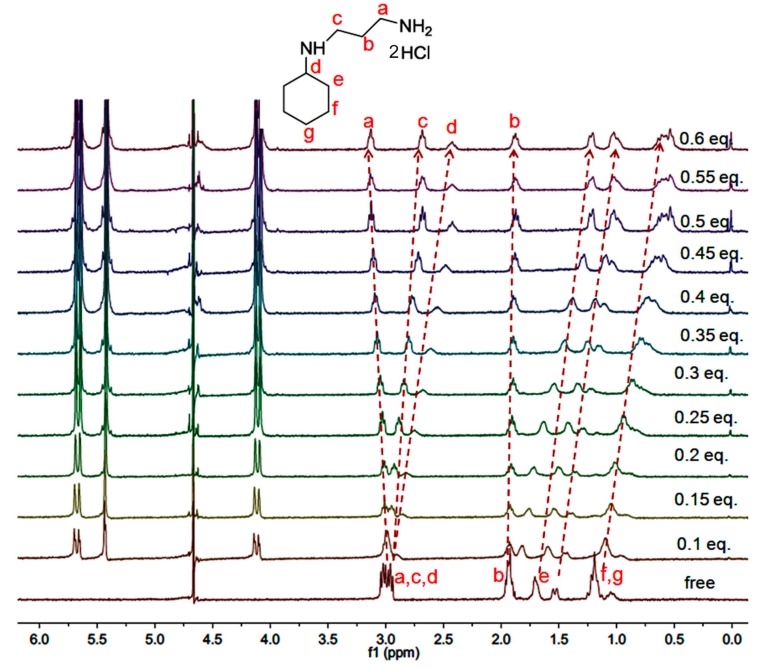
^1^H NMR titration spectra of the *N*-(3-aminopropyl)cyclohexylamine (N3ACyA) guest in D_2_O (1.0 mmol, pD = 2.45) with increasing concentrations of the Q[8] host at room temperature.

As shown in Scheme 1, four cyclohexylamine derivatives (hydrochloride salts), namely cyclohexylamine (CyA), *N*-ethylcyclohexylamine (NECyA), *N*-cyclohexylethanolamine (NCyEA), and *N*-(3-aminopropyl)cyclohexylamine (N3ACyA), were selected as the guests for binding to the Q[8] host. ^1^H NMR titration experiments clearly indicated that the cyclohexyl moieties on these guests can be included into the cavity of the Q[8] to form host-guest interactions (Figures S1–S3). Figure 1 shows the detailed ^1^H NMR spectra of the N3ACyA guest in D_2_O (pD = 2.45), which was recorded in the absence and presence of different concentrations of Q[8]. The free N3ACyA guest peaks were well-resolved when no Q[8] was added. After the addition of the Q[8] host to the guest solution, new peaks appeared at 4.10 ppm, 5.43 ppm and 5.67 ppm, which are ascribed to Q[8] (Figure 1). Upon increasing the molar ratio of Q[8], the resonances for the cyclohexylamine protons exhibited a significant shift to a higher field. In particular, the peaks associated with the protons on the aminopropyl moiety underwent an interesting chemical shift. For example, protons H_a_ and H_c_ on the aminopropyl moiety are buried together with proton H_d_ on the cyclohexylamine group around *δ* 3.0 ppm in the free state of the guest, and are very difficult to distinguish. However, in the presence of the Q[8] host, the resonances of such proton peaks are well resolved with distinct chemical shifts. For example proton H_a_ is shifted downfield, whereas protons H_c_ and H_d_ undergo larger upfield shifts. On the other hand, no obvious change in the chemical shift of proton H_b_ on the cyclohexylamine group was observed. The upfield proton chemical shift is due to the shielding effect of the hydrophobic cavity, and the relative downfield shift is due to the deshielding effect of the carbonyl-rimmed portal of the Q[8] host. In other words, these ^1^H NMR spectroscopic results indicate that the cycloalkane moiety of the guest is deeply encapsulated within the cavity of the Q[8] host, and that most of the aminopropyl moiety is located at the portal of the Q[8] host. Notably, all of the protons on the guest remain in the same area when the concentrations of Q[8] that were added to the guest solution increased above 0.5 equiv., indicating that that the Q[8] host can accommodate two cycloalkane moieties within the same cavity to form a 1:2 ternary host-guest inclusion complex.

**Figure 2:**
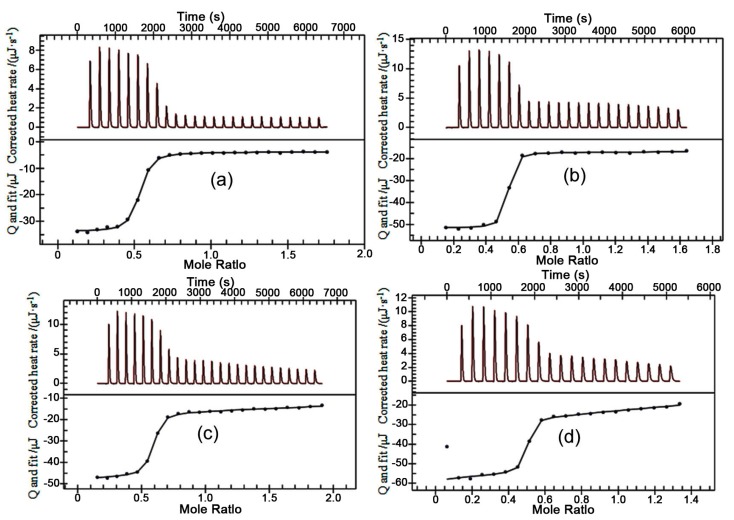
Isothermal titration calorimetry (ITC) data for the complexation of Q[8]·cyclohexylamine (CyA) (**a**); Q[8]·*N*-ethylcyclohexylamine (NECyA) (**b**); Q[8]·*N*-cyclohexylethanolamine (NCyEA) (**c**); Q[8]·N3ACyA (**d**) at 298.15 K (pH 2.45).

In supramolecular chemistry, isothermal titration calorimetry (ITC) is a useful tool for monitoring host−guest interactions. For example, ITC can be employed to quantify the enthalpic and entropic contributions to the binding interactions between the hosts and guests. Herein, as shown in Figure 2 and Table 1, the thermodynamic parameters obtained from the ITC experiments suggest that the host–guest interactions of Q[8] with these cyclohexyl appended guests are driven by both enthalpy and entropy factors. Furthermore, by fitting the data, a host-guest complex molar ratio of 1:2 for Q[8] with the guests is also confirmed. For example, the complexation of guest N3ACyA with the Q[8] host is driven by both enthalpy and entropy (Δ*H*° = −26.77 kJ·mol^−1^ and *T*Δ*S*° = 14.26 kJ·mol^−1^). The binding constant (*K*_a_) for the complexation was determined to be 1.55 × 10^7^ M^−1^ at 298.15 K, which is much larger than that of previously reported ternary complexes involving alkyl groups inside the Q[8] cavity (*K*_a_ on the order of 10^5^ M^−1^) [18]. In such cases, alkyl groups such as a butyl were appended to a 4,4′-bipyridinium core, and the resulting ternary complex most probably stemmed from the contribution of the ion–dipole interactions between the positively charged nitrogen atoms of the 4,4′-bipyridinium and the Q[8] portal carbonyl oxygen atoms. The ITC data indicated that the Q[8] cavity-based ternary complex involving an alkyl group is mostly enthalpy driven. In the present study, as mentioned above, the host-guest interactions are driven by both enthalpy and entropy factors. Negative enthalpy changes may result from the ion–dipole interactions between the protonated nitrogen atoms on the guest, the carbonyl oxygen atoms at the portals of the Q[8] cavity, and the non-classical hydrophobic effect from the desolvation of the Q[8] host cavity releasing ‘high-energy’ water trapped in the cavity. The positive entropy is mainly derived from the classical hydrophobic effect of the guest, such as the cyclohexyl moiety in aqueous solution. Notably, the binding constants for these four inclusion complexes are in the range of 10^6^~10^7^ M^−1^, indicating that the cyclohexyl-based ternary complex inside the Q[8] cavity is more stable compared with an alkyl moiety, and even with aromatic groups [6]. For example, it is reported that the Q[8]·MV^2+^ (methylviologen) can further bind a variety of aromatic electron-donating guests with binding constants that range from 10^2^~10^6^ M^−1^ [2].

**Figure 3:**
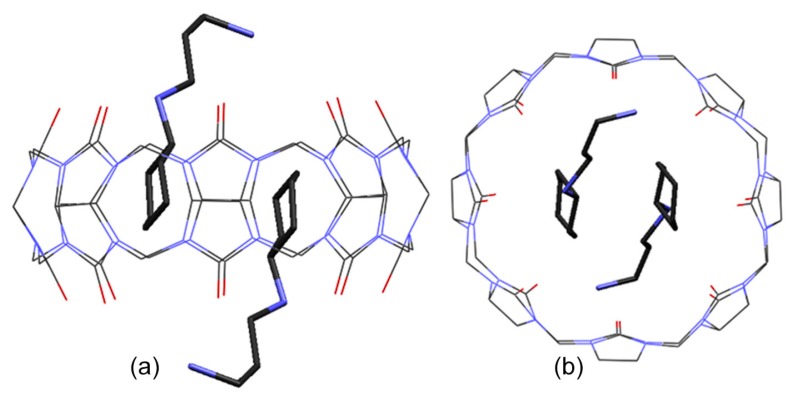
X-ray strucure of Q[8]·N3ACyA: (**a**) side view; (**b**) top view. Hydrogen atoms, solvate water molecules, and [CdCl_4_]^2^^−^ dianions are omitted for clarity.

In an effort to gain more detailed binding information on the host-guest interactions between Q[8] and the cyclohexyl-appended guests, we successfully obtained the X-ray crystal structure of Q[8]·N3ACyA. The slow evaporation of a hydrochloric acid aqueous solution containing a 1:2 mixture of the Q[8] host and N3ACyA in the presence of CdCl_2_ resulted in colorless crystals of the inclusion complex Q[8]·N3ACyA at room temperature. As shown in Figure 3, the crystal structure analysis revealed that two cyclohexylamine moieties on the guest were included inside the cavity of the Q[8] host, and positioned in opposing orientations related by the inversion centre. Each of these is on average half occupied. The aminopropyl group was found to reside outside of the two portals of the Q[8] host, which is in agreement with the solution behavior observed by ^1^H NMR spectroscopy. The shortest distance between the two encapsulated cyclohexylamine moieties in the Q[8] cavity is around 2.28 Å. Notably, apart from the ion-dipole interactions between the ammonium guest and the carbonyl groups of the host portal, it is evident that the presence of each guest is further stabilized by the C−H···π interactions (bond distances in the range 3.131 Å–3.368 Å) between the included cycloalkane and C=O group of the Q[8] host. Hydrogen bonding between the outside terminal ammonium group of the guest and the carbonyl-laced portal of the Q[8] host will also contribute to the formation of the ternary inclusion complex of Q[8]·N3ACyA.

## 3. Materials and Methods

### 3.1. General

Cyclohexylamine, *N*-Ethylcyclohexylamine, *N*-Cyclohexylethanolamine, and *N*-(3-Aminopropyl)-cyclohexylamine were purchased from TCI (Shanghai, China) Development Co., Ltd. Other chemicals were commercially available and used as received without further purification.

### 3.2. Isothermal Titration Calorimetry (ITC)

ITC measurements were carried out using a Nano ITC (New Castle, TA, USA) isothermal titration calorimeter at 298.15 K. The heats of reaction were corrected for the heat of dilution of the guest solution determined in separate experiments. All of the solutions were degassed prior to titration by sonication. Computer simulations were performed using the Nano ITC analyze software.

### 3.3. Crystal Structure Determination

A suitable single crystal (0.2 × 0.2 × 0.1 mm^3^) was taken up in paraffin oil and mounted on a Bruker SMART Apex II CCD diffractometer (Bruker AXS, Madison, WI, USA) equipped with a graphite monochromated Mo-K_α_ radiation source (λ = 0.71073 Å, μ = 0.828 mm^−1^) operating in the ω-scan mode. Data were corrected for Lorentz and polarization effects using the program SAINT (Bruker AXS, Madison, WI, USA). The structure was solved by direct methods, and was refined against F^2^ using the full-matrix least-squares method using SHELXL-2016 [19]. Data beyond 0.9 Å resolution were omitted from the refinement, as these are essentially noise. Non-hydrogen atoms were refined anisotropically. Small-scale disorder in the position of the anions was modeled using standard techniques. Carbon-bound hydrogen atoms were introduced at calculated positions, and were treated as riding atoms with an isotropic displacement parameter equal to 1.2 times that of the parent atom. The water molecules in the compounds were omitted by using the SQUEEZE option of the PLATON program. Crystallographic data for the reported structures have been deposited at the Cambridge Crystallographic Data Centre as supplementary publication nos. CCDC-1587234. These data may be obtained free of charge via http://www.ccdc.cam.ac.uk/data_request/cif, by emailing data_request@ccdc.cam.ac.uk, or by contacting The Cambridge Crystallographic Data Centre, 12, Union Road, Cambridge CB2 1EZ, UK (Fax: +44 1223 336033).

Crystal data for the host-guest complex: 2(C_48_H_46_N_32_O_16_)·C_9_H_22_N_2_·2(CdCl_4_)·[10H_2_O], *M*_r_ = 3321.02, triclinic, space group P-1, a = 17.499(12) Å, b = 17.654(12) Å, c = 18.005(13) Å, *α* = 70.891(9)^o^, *β* = 81.545(10)^o^, *γ* = 65.968(9)^o^, *V* = 4799(6) Å^3^, Z = 1, *D*_c_ = 1.149 g cm^−^^3^, F(000) = 1694, *R*_1_ = 0.0944 (*I* > 2*σ*(*I*)), w*R*_2_ = 0.2930 (all data), GoF = 0.985.

Anal. Calcd. for C_105_H_114_N_66_O_3__2_ Cd_2_Cl_8_ (%): C 37.98, H 3.46, N 27.84; found: C 36.91, H 3.85, N 26.96.

## 4. Conclusions

In summary, the selective host-guest binding behavior of Q[8] with a series of cyclohexyl group-appended guests has been evaluated by ^1^H NMR spectroscopy, ITC, and X-ray crystallography. All of the results suggested that the Q[8] host can simultaneously hold two cyclohexyl in the hydrophobic cavity. In particular, the solid state structure of the ternary complex suggests that the high binding constant of Q[8]·N3ACyA likely results from the multiple cooperation of the ion-dipole interactions, hydrophilic effects, and C–H···π interactions between the guest and the host.

## Figures and Tables

**Table 1:** Thermodynamic binding data for Q[8]·CyA, Q[8]·NECyA, Q[8]·NCyEA, Q[8]·N3ACyA.

Host-Guest	*K*a/(L·mol^−1^)	*n*	Δ*G*/(kJ·mol^−1^)	Δ*H*/(kJ·mol^−1^)	*T*Δ*S*/(kJ·mol^−1^)
Q[8]·CyA	(3.07 ± 0.02) × 10^6^	0.504	−37.02 ± 0.2	−29.48 ± 0.2	7.54 ± 0.4
Q[8]·NECyA	(1.27 ± 0.04) × 10^7^	0.500	−40.55 ± 0.6	−33.96 ± 0.3	6.59 ± 0.3
Q[8]·NCyEA	(4.59 ± 0.03) × 10^6^	0.554	−37.93 ± 0.4	−28.67 ± 0.3	9.26 ± 0.2
Q[8]·N3ACyA	(1.55 ± 0.02) × 10^7^	0.475	−41.03 ± 0.4	−26.77 ± 0.2	14.26 ± 0.3

## Supplementary Materials

The supplementary materials are available online.
