# Supplementary material for: Host-Guest Interaction of Cucurbit[8]uril with N-(3-Aminopropyl)cyclohexylamine: Cyclohexyl Encapsulation Triggered Ternary Complex
Source: Molecules. 2018 Jan 15;23(1):175. doi: 10.3390/molecules23010175 (PMC6017755; doi:10.3390/molecules23010175)
Supplement: Supplementary file 1 [file molecules-23-00175-s001.pdf]

Supporting information

Host-guest interaction of cucurbit[8]uril with N-(3-Aminopropyl)cyclohexylamine: cyclohexylalkane encapsulation triggered ternary complex

Yu Xia[1], Chuan-Zeng Wang[1], Mengkui Tian[1], Zhu Tao[1], Xin-Long Ni[1*], Timothy J. Prior[2] and Carl Redshaw[2*]

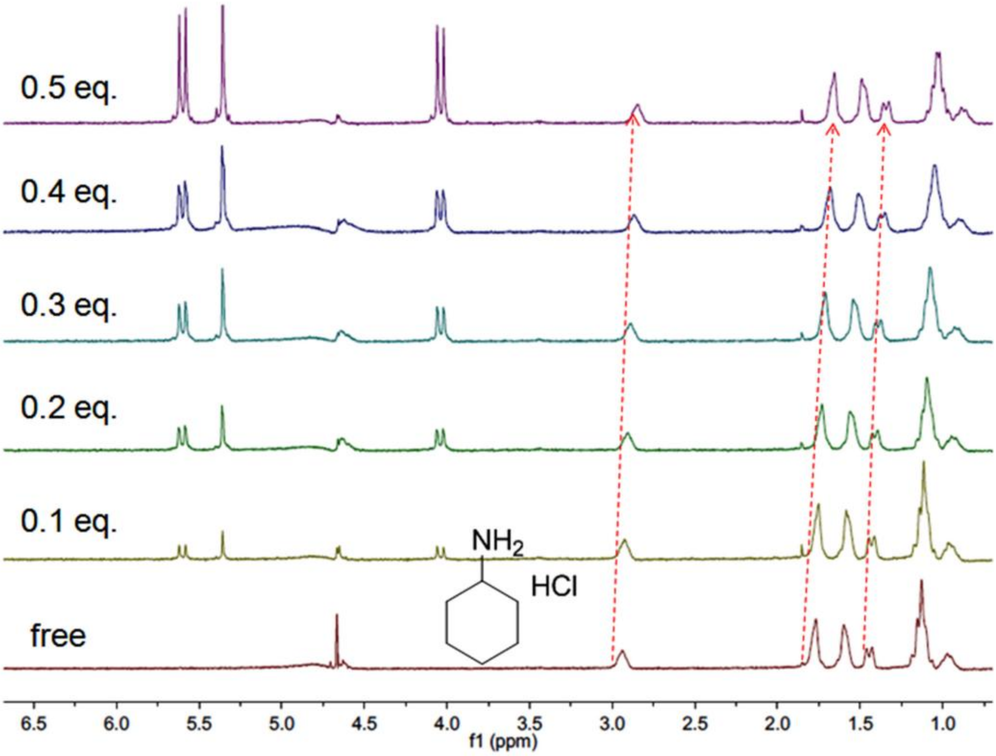

Figure S1. ^{1}H NMR titration spectra of the **CyA** guest in D_2O (1.0 mmol, pD = 2.45) with increasing concentrations of the Q[8] host at room temperature.

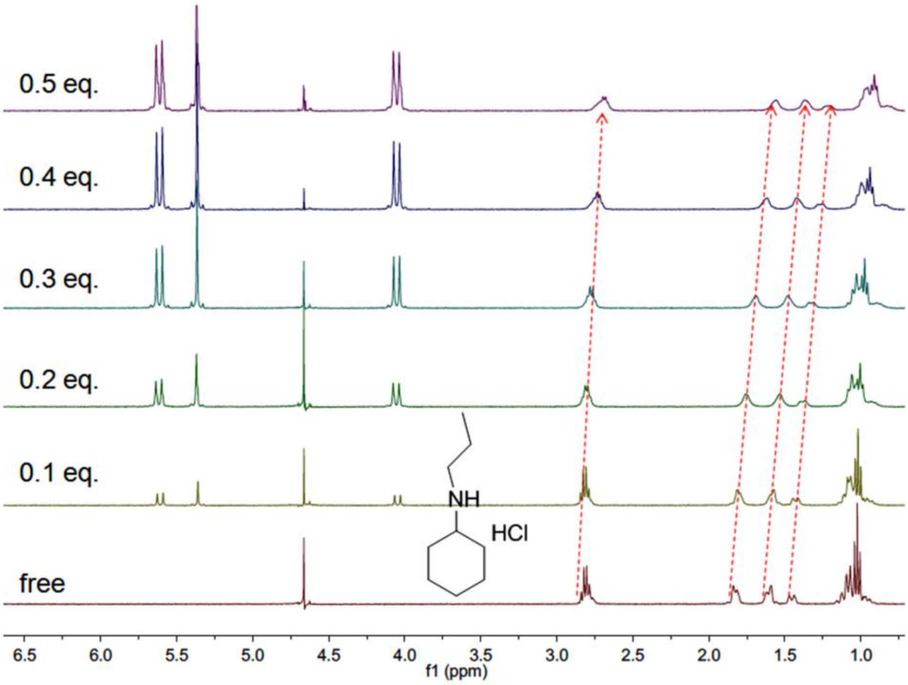

Figure S2. ^{1}H NMR titration spectra of the **NECyA** guest in D_2O (1.0 mmol, pD = 2.45) with increasing concentrations of Q[8] host at room temperature.

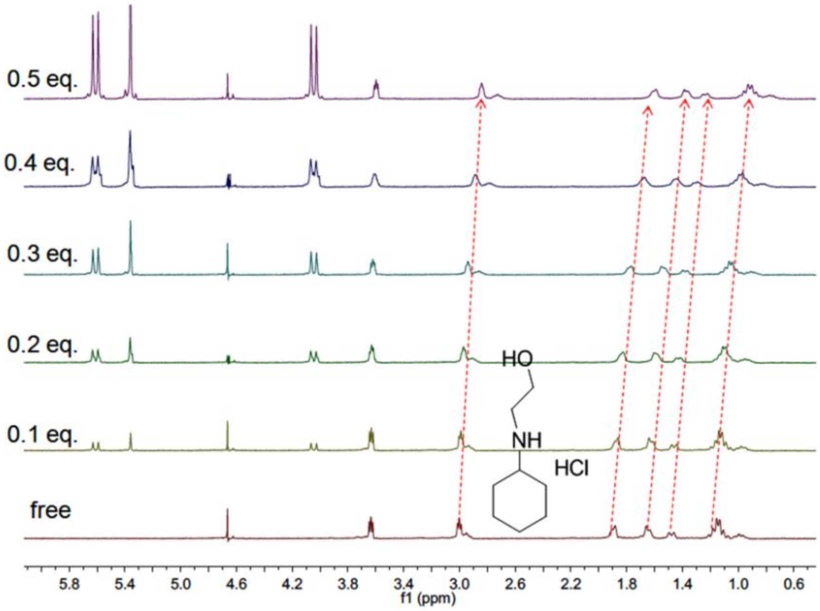

Figure S3. ^{1}H NMR titration spectra of the **NCyEA** guest in D_2O (1.0 mmol, pD = 2.45) with increasing concentrations of the Q[8] host at room temperature.

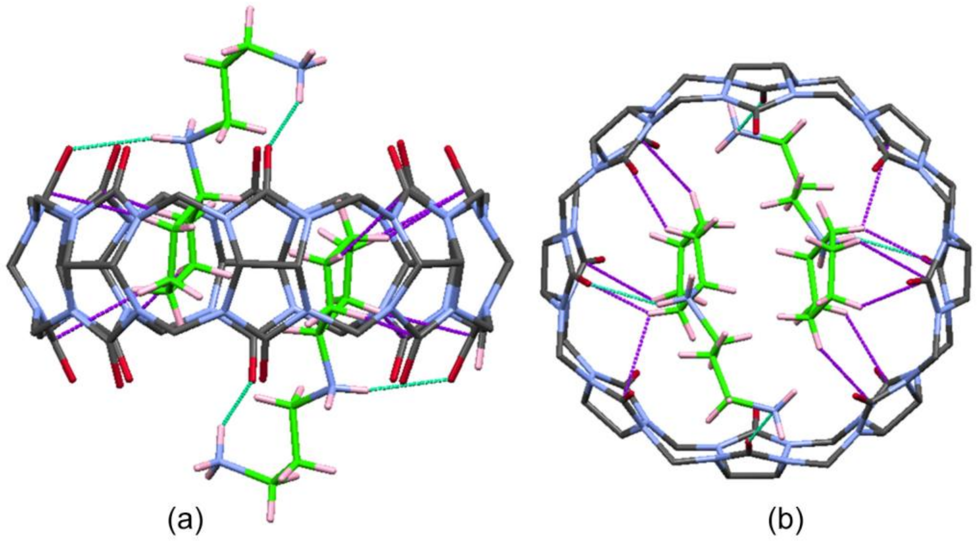

Figure S4. X-ray strucure of Q[8]•**N3ACyA**: (a) side view; (b) top view; hydrogen bond (green), C−H···π interaction (magenta). Hydrogen atoms, solvate water molecules, and $[CdCl_4]^{2-}$ dianions are omitted for clarity.
